# Pathway-specific polygenic risk scores for blood pressure traits in a West African cohort

**DOI:** 10.3389/fcvm.2026.1707809

**Published:** 2026-03-13

**Authors:** Gregory Bormes, Vanessa Robbin, Tinashe Chikowore, Yuji Zhang, Ananyo Choudhury, Scott Hazelhurst, Neil A. Hanchard, Sally N. Adebamowo, Adebowale A. Adeyemo, Bamidele Tayo

**Affiliations:** 1 Stritch School of Medicine, Loyola University Chicago, Maywood, IL, United States; 2 Harvard Medical School, Boston, MA, United States; 3 Channing Division of Network Medicine, Brigham and Women’s Hospital, Boston, MA, United States; 4 Department of Epidemiology and Public Health, University of Maryland School of Medicine, Baltimore, MD, United States; 5 Sydney Brenner Institute for Molecular Bioscience, University of the Witwatersrand, Johannesburg, South Africa; 6 School of Electrical and Information Engineering, University of the Witwatersrand, Johannesburg, South Africa; 7 Center for Precision Health Research, National Human Genome Research Institute, National Institutes of Health, Bethesda, MD, United States; 8 Greenebaum Comprehensive Cancer Center, University of Maryland School of Medicine, Baltimore, MD, United States; 9 Center for Research on Genomics and Global Health, National Human Genome Research Institute, National Institutes of Health, Bethesda, MD, United States; 10 Department of Public Health Sciences, Parkinson School of Health Sciences and Public Health, Loyola University Chicago, Maywood, IL, United States

**Keywords:** antihypertensive agents, blood pressure traits, genome-wide association study (GWAS), pathway-specific, polygenic risk scores

## Abstract

**Introduction:**

Genome-wide polygenic risk scores (PRSs) are useful for stratifying individuals' risk of polygenic diseases such as hypertension. However, a limitation of genome-wide PRSs is that they do not provide information about the distribution of risk burden across biological pathways. We used pathway-specific PRSs to investigate these effects of common antihypertensive therapy target pathways on disease risk in a cohort of West African individuals.

**Methods:**

A total of 11 pathways, comprising 1,149 unique genes, were selected based on the targets of commonly used antihypertensive agents. Pathway-specific PRSs for hypertension [individuals with systolic blood pressure (SBP) ≥140 mmHg, diastolic blood pressure (DBP) ≥90 mmHg, or taking antihypertensive medications] were calculated in a cohort of 2,295 individuals. The model was then validated and tested in independent cohorts of 1,614 and 966 individuals, respectively. All participants were recruited from the International Collaborative Study on Hypertension in Blacks.

**Results:**

In the combined pathway analysis, PRSs predicted risk better than base models fitted only with sex, age, and principal components. Compared with the base models without the PRSs, incremental increases in *R*^2^ attributable to the inclusion of PRSs in predictive models were 2.6% for SBP (*p* = 0.009), 1.4% for DBP (*p* = 0.012), and 1.1% for mean arterial pressure (MAP) (*p* = 0.044). PRSs from certain signaling pathways (mitogen-activated protein kinase, cAMP, and adrenergic signaling in cardiomyocytes) could stratify individuals into the top and bottom deciles of DBP risk. Adrenergic signaling in cardiomyocytes was also predictive of MAP when comparing individuals in the top and bottom deciles.

**Conclusions:**

Combined pathway polygenic risk scores derived from genes in well-defined genetic pathways predict hypertension risk in individuals of African ancestry. However, the relatively low predictability of pathway-specific PRSs supports the need to explore the broader influence of genetic, environmental, and epigenetic factors that cannot be captured by pathway-specific PRSs alone.

## Introduction

The growing prevalence of hypertension across populations represents a global health concern, with an estimated 1.28 billion individuals currently impacted and fewer than one-quarter of those considered controlled (1). In African populations, recent large-scale epidemiological studies have shown that hypertension affects approximately 28%–30% of adults, with substantial regional variation and concerning gaps in awareness, treatment, and control (2–5). Hypertension carries a significant mortality risk, and uncontrolled hypertension is associated with significant sequelae, including stroke and myocardial infarction. Despite the long history of antihypertensive therapy, affected individuals frequently respond unpredictably and/or ineffectively to its treatment (6). Understanding the genetic contributions to the progression of this multifactorial disease has been an important area of research in cardiovascular medicine (7, 8). Improving our understanding of the mechanisms underlying hypertension pathophysiology may enable clinicians to better identify patients at high risk, facilitate closer monitoring, and guide the use of more targeted therapies.

Genome-wide polygenic risk scores (PRSs) are a promising tool for stratifying individual disease risk by aggregating the effects of hundreds or thousands of genes that often span the entire genome (8). While the traditional genome-wide approach has the potential to better characterize highly polygenic traits by identifying unexpected contributors to certain traits, it has some associated drawbacks. For traits and disorders that involve dysregulation across multiple systems and pathways, such as hypertension, a key limitation of PRSs is that they offer little insight into how overall genetic risk is apportioned across distinct biological pathways. This can limit understanding of the underlying mechanisms of disease and thus limit the interpretability of PRSs. Narrowing the scope of PRS analyses to putative biological pathways can offer more specific insights into disease mechanisms, individual risk profiles, and medication efficacy (7, 9). Accordingly, in recent years, there has been strong interest in pathway-specific polygenic risk scores across a wide variety of conditions, including coronary artery disease, Alzheimer's disease, and Parkinson's disease (9–12). Notably, pathway-specific PRS have outperformed conventional PRS in stratifying individuals by disease risk (10, 13, 14).

Many commonly used antihypertensive medications act on specific, well-defined biological pathways (6). Previous pathway-based PRS studies conducted in European cohorts have identified several genetic pathways associated with hypertension; however, their findings have shown limited generalizability to other populations (7). In particular, the predictive performance of genome-wide PRSs has been consistently lower in African and African-ancestry populations than in European populations. For example, a PRS for hypertension explained 8.0% of the variance in systolic blood pressure (SBP) and 7.8% in diastolic blood pressure (DBP) among White individuals compared with 3.5% and 3.1% among Black individuals, respectively (8). In a cohort of 5,200 sub-Saharan Africans and 55,034 SNPs, PRSs explained only 1.2% of the variance in diastolic blood pressure and 0% in systolic blood pressure (15). In another study in a cohort of 10,602 African individuals with hypertension, PRSs explained 1.31% of the variance in systolic blood pressure and 1.10% of the variance in diastolic blood pressure (16). Also, a recent study by Onyenobi et al. demonstrated that the predictive accuracy of genome-wide PRSs for blood pressure (BP) traits varied across different regions of Africa (17).

The need for improved genetic risk stratification in African populations is underscored by the substantial and growing burden of hypertension across the continent. A recent systematic review and meta-analysis encompassing over 1 million individuals from multiple African regions reported high prevalence of hypertension, accompanied by low levels of awareness, treatment, and control, emphasizing the urgent public health relevance of ancestry-specific hypertension research (5).

Given the poor performance of existing genome-wide PRS models in African-ancestry populations, we investigated how genetic variation within genes constituting common antihypertensive medication target pathways affects hypertension risk in a cohort of Africans residing in Nigeria (8, 18). We calculated pathway-specific PRSs for hypertension using three separate cohorts from the same homogeneous ancestry group to create, validate, and test the model. The pathways of interest were selected based on targets of common first-line antihypertensive medications (Table 1) (6).

**Table 1:** Target pathways of common antihypertensive medications.

Pathway	KEGG ID	Number of genes
Adrenergic signaling in cardiomyocytes^a^^,^^b^^,^^c^	hsa04261	170
Aldosterone synthesis and secretion^a^	hsa04925	116
Calcium signaling^a^^,^^b^^,^^c^	hsa04020	269
Cardiac muscle contraction^b^^,^^c^^,^^d^	hsa04260	93
MAPK signaling pathway^b^	hsa04010	343
Neuroactive ligand receptor interaction^a^^,^^c^	hsa04080	392
Renin–angiotensin system^a^^,^^d^	hsa04614	24
Renin secretion^a^	hsa04924	79
cGMP–PKG signaling pathway^c^	hsa04022	183
cAMP signaling pathway^c^	hsa04024	245
Vascular smooth muscle contraction^a^^,^^b^	hsa04270	146

Biological pathways used to construct pathway-specific polygenic risk scores (PRSs) for hypertension (HTN) are shown. Pathway definitions and gene membership were obtained from the Kyoto Encyclopedia of Genes and Genomes (KEGG). Each pathway is annotated with its corresponding KEGG identifier and the total number of genes included. Superscripts indicate the classes of antihypertensive medications whose known pharmacologic targets map to genes within each pathway:

^a^ Angiotensin receptor blockers.

^b^ Calcium channel blockers.

^c^ Beta-blockers.

^d^ Angiotensin–converting-enzyme inhibitor.

These pathways were selected *a priori* based on established mechanisms of action of common HTN therapies.

## Methods

### Study participants

The data analyzed in this study were from three non-overlapping Nigerian cohorts from the same homogeneous ancestry group, whose sampling frames were provided by the International Collaborative Study on Hypertension in Blacks (ICSHIB), as described in detail elsewhere (19, 20). The ICSHIB base sample included more than 15,000 participants recruited from the Yoruba-speaking city of Ibadan and the town of Igbo-Ora in Oyo State, Nigeria. From this base sample, three non-overlapping cohorts, namely, NEX1KG (*n* = 2,295), NGR1KG (*n* = 1,614), and NAX1KG (*n* = 966), with available genome-wide genotype data were included in the present study. The project was reviewed and approved by Loyola University Chicago and the University of Ibadan. All participants signed informed consent, administered in either English or Yoruba.

### Phenotype measurement

A screening examination was performed by trained research staff using a standardized protocol (21). Information on family and medical history was obtained from each participant. BP measurements were conducted by staff trained and certified according to a previously described procedure (19, 21). An oscillometric device, which had previously been evaluated in similar field settings, was used for all BP measurements (21). Three measurements were obtained at 3-min intervals, and the average of the final two was used for analysis. Individuals with SBP ≥140 mmHg, DBP ≥90 mmHg, or on antihypertensive medication at the time of examination were defined as hypertensive. Participants identified as hypertension by these criteria were referred for treatment and subsequently followed up by a physician.

### Genotyping and imputation quality assessment

Genome-wide SNP array data from the three cohorts underwent quality control procedures, as described previously by Nandakumar et al. (22). Genotyping for the cohorts was performed using the Illumina Human Exome BeadChip v1.0 (NEX1KG cohort, *n* = 2,295), the Affymetrix Genome-Wide Human SNP Array 6.0 (NGR1KG cohort, *n* = 1,614), and the Affymetrix Axiom Genome-Wide KP UCSF AFR Array (NAX1KG cohort, *n* = 966), yielding 162,779, 723,995, and 803,260 genotyped variants, respectively, after applying standard genome-wide association study (GWAS) quality control filters and prior to imputation. Using the Sanger Imputation Service, we performed genotype imputation separately for each cohort to infer missing genotypes or genotypes at ungenotyped loci using the 1000 Genomes Phase 3 Reference Panel, yielding 85 million imputed variants in each cohort (23, 24). Standard postimputation quality control filters were applied to retain typed variants and exclude failed imputed variants, multiallelic variants, and variants with an imputation quality value less than 0.3 or a minor allele frequency less than 0.05. The total number of variants remaining after postimputation quality control procedure were 751,845 for NEX1KG, 6,729,356 for NGR1KG, and 9,607,460 for NAX1KG.

### Pathway selection and SNP mapping

Biological pathways were selected based on their known involvement in antihypertensive medication mechanisms. The KEGG Pathway Database was used to identify and select pathways targeted by common first-line antihypertensive medications. Genetic pathway targets of beta-blockers, calcium channel blockers, angiotensin–converting-enzyme inhibitor (ACEIs), and angiotensin receptor blockers were included (Table 1). The selected pathways represent key processes in blood pressure pathophysiology, regulation, and therapeutic response.

For each pathway, we extracted single-nucleotide polymorphisms (SNPs) available in our imputed GWAS datasets by mapping SNPs to pathway genes based on coordinates from the Genome Reference Consortium Human Build 37.

### Cohort specification

Three independent cohorts served as discovery, validation, and target cohorts (19, 20). The discovery cohort (NEX1KG, *n* = 2,295) was used for initial SNP-BP associations testing and effect size estimation. The validation cohort (NGR1KG, *n* = 1,614) was used for PRS optimization and threshold selection. The target cohort (NAX1KG, *n* = 966) was used to assess the performance of the PRS model.

#### PRS calculation and analysis

##### Discovery cohort

Polygenic risk scores were calculated for SBP, DBP, mean arterial pressure (MAP), and pulse pressure (PP) for each of the 11 target pathways. In addition, a combined-pathway PRS was calculated for each trait using genes aggregated from all the pathways.

Analyses of SNP-BP associations in the NEX1KG discovery cohort were performed for each BP measure as a continuous variable by fitting an additive genetic model for each SNP in a linear regression model adjusted for age and sex. Adjusted BP measures were used for individuals on antihypertensive medications by adding 10 and 15 mmHg to measured diastolic BP and systolic BP, respectively, according to a standard method used in BP GWAS (25, 26). The first five genetic principal components were also included as covariates to correct for effects of any potential population stratification (27). For each SNP, the risk allele was defined as the trait-increasing allele, and every SNP was coded as the count or dosage of the respective risk allele per genotype. Training of the PRS model was performed on the discovery cohort using the “clumping and thresholding” method (28).

To address linkage disequilibrium within the cohort, clumping was performed using PLINK version 1.9 in the independent NGR1KG validation cohort, which served as the LD reference panel (29, 30). LD was therefore estimated within the study cohort rather than using an external reference panel, thereby ensuring ancestry matching between LD estimation and PRS evaluation. SNPs within 250 kb of the index SNP were considered for clumping. SNPs with *r*^2^ > 0.1 based on maximum likelihood haplotype frequency estimates were considered to be in linkage disequilibrium with the index SNP and were thus excluded from analysis.

PRSs were calculated for combined pathways at seven *p*-value thresholds (0.0010, 0.0025, 0.0050, 0.0075, 0.0100, 0.0250, and 0.0500), and corresponding effect sizes for the SNPs were calculated and extracted for use as weights in the validation and target cohorts. In addition, pathway-specific PRSs were calculated at each threshold. We used the default settings for PRS calculation, as implemented in PLINK (30).

##### Validation cohort

We used the NGR1KG validation cohort to identify the best-fitting PRS model by fitting a linear regression model of BP traits on PRS at seven different *p-*value thresholds, with sex, age, and the top five principal components included as covariates (22). The best-fit *p-*value threshold was chosen to maximize the incremental *R*², calculated as $R_{\mathrm{increment}}^{2}\;=\;R_{\mathrm{full}}^{2}-R_{\mathrm{base}}^{2}$, where *R*²_full_ includes PRS and covariates, while *R*²_base_ includes only covariates.

##### Target cohort

To assess the predictive performance of the selected PRS model for the respective BP traits in both individual and combined pathways, we applied the PRS model to the independent NAX1KG target cohort. Performance was evaluated using incremental *R*², as in the validation cohort. Predictive performance was assessed using covariate-adjusted linear regression models. PRS effect estimates, standard errors, *p*-values, and incremental *R*² values were reported. Samples in the target cohort were divided into deciles based on PRS values. The mean and standard deviation (SD) of each decile were calculated for each BP trait. To determine whether PRSs could reliably differentiate among mean BP traits, the 1st and 10th deciles were compared using a two-sample *t*-test.

### Quality control and software

All analyses were performed using R version 4.3.3 for statistical computing and PLINK versions 1.9 and 2.0 for genetic analyses (30). Quality control measures included assessment of genotyped SNP missingness, minor allele frequency, and Hardy–Weinberg equilibrium. Intermediate results, including SNP lists, clumping results, combined pathways, and pathway-specific PRS distributions, were documented and archived for reproducibility. Phenotype data were adjusted for age, sex, and the top five principal components to account for potential confounding factors and/or population stratification. The exact PLINK commands used for LD clumping and PRS scoring are provided in a GitHub repository as part of the Supplementary Methods (31).

## Results

### Cohort Characteristics

The descriptive characteristics of individuals in the discovery, validation, and target cohorts are presented in Table 2. The discovery cohort included 2,295 individuals with a mean age of 48.7 ± 11.2 years, mean SBP of 159.7 ± 29.5 mmHg, and mean DBP of 101.3 ± 18.2 mmHg. The validation cohort contained 1,614 individuals with a mean age of 49.0 ± 14.5 years, mean SBP of 135.4 ± 29.9 mmHg, and mean DBP of 83.7 ± 18.6 mmHg. Similarly, the target cohort contained 966 individuals with a mean age of 53.1 ± 11.5 years, mean SBP of 152.0 ± 31.5 mmHg, and mean DBP of 93.6 ± 19.3 mmHg.

**Table 2:** Study population characteristics.

Variable	Discovery cohort (NEX1KG)		Validation cohort (NGR1KG)		Target cohort (NAX1KG)	
	*N*	Mean (SD) or *n* (%)	*N*	Mean (SD) or *n* (%)	*N*	Mean (SD) or *n* (%)
Age (years)	2,295	48.7 (11.2)	1,614	49.0 (14.5)	966	53.1 (11.5)
Female, *n* (%)	1,683	73.3%	940	58.2%	734	76%
Male, *n* (%)	612	26.7%	674	41.8%	232	24%
SBP (mmHg)	2,295	159.7 (29.5)	1,614	135.4 (29.9)	966	152.0 (31.5)
DBP (mmHg)	2,295	101.3 (18.2)	1,614	83.7 (18.0)	966	93.6 (19.3)
PP (mmHg)	2,295	58.4 (17.0)	1,614	51.7 (16.4)	966	58.4 (17.1)
MAP (mmHg)	2,295	120.7 (21.1)	1,614	100.9 (21.7)	966	113.1 (22.6)

Baseline demographic and clinical characteristics of the discovery (NEX1KG), validation (NGR1KG), and target (NAX1KG) cohorts are shown. Continuous variables are summarized as mean and standard deviation (SD), and categorical variables are presented as counts and percentages. Blood pressure-related phenotypes include systolic blood pressure (SBP), diastolic blood pressure (DBP), pulse pressure (PP), and mean arterial pressure (MAP). These cohorts were used sequentially for PRS creation, validation, and testing to assess the performance of pathway-specific polygenic risk scores for hypertension.

### Combined-pathway PRSs

Compiling genes across all 11 pathways yielded a list of 1,149 unique genes, with a total of 13,237 SNPs present across the discovery, validation, and target cohorts. This gene set was used to calculate the combined-pathway PRSs for SBP, DBP, MAP, and PP.

After clumping, 1,154 independent SNPs remained for the SBP PRS calculation. The optimal *p-*value threshold for SBP was 0.05, yielding a list of 101 SNPs for analysis, corresponding to 85 unique genes. In our target cohort, the incremental increase in *R*^2^ due to the SBP PRS was 0.0056 (2.6%, *p* = 0.009).

**Figure 1:**
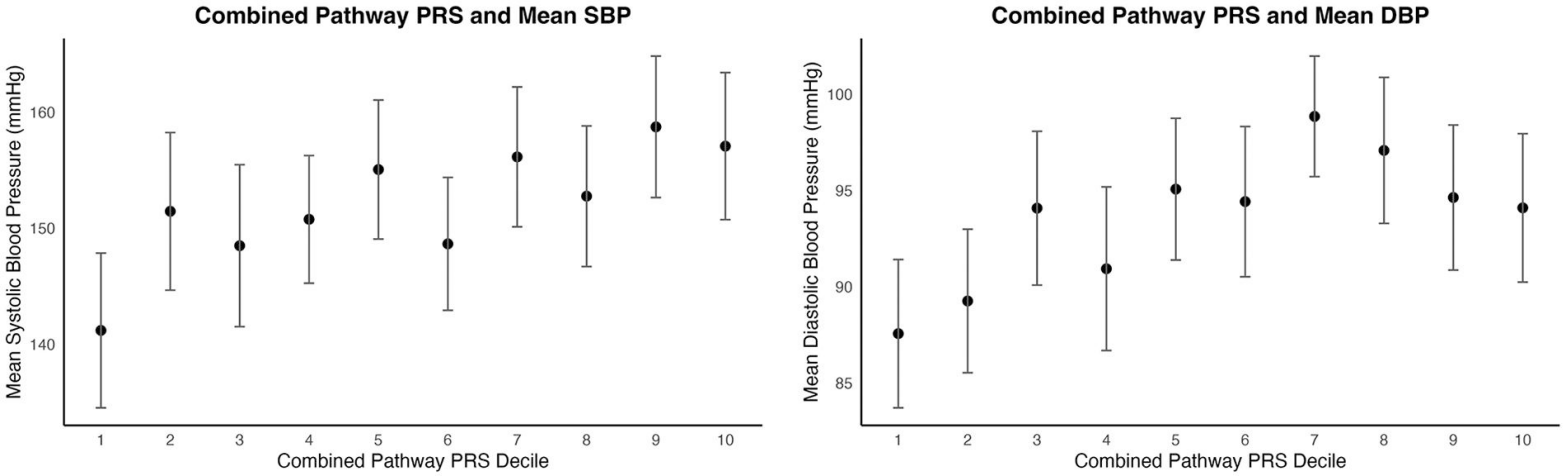
Association between the aggregate pathway polygenic risk score (PRS) and blood pressure traits for SBP (left) and DBP (right). (Left) Mean systolic blood pressure (SBP) and (right) mean diastolic blood pressure (DBP) across deciles of the aggregate pathway PRS. Points denote decile-specific means, and standard error bars represent 95% confidence intervals.

**Figure 2:**
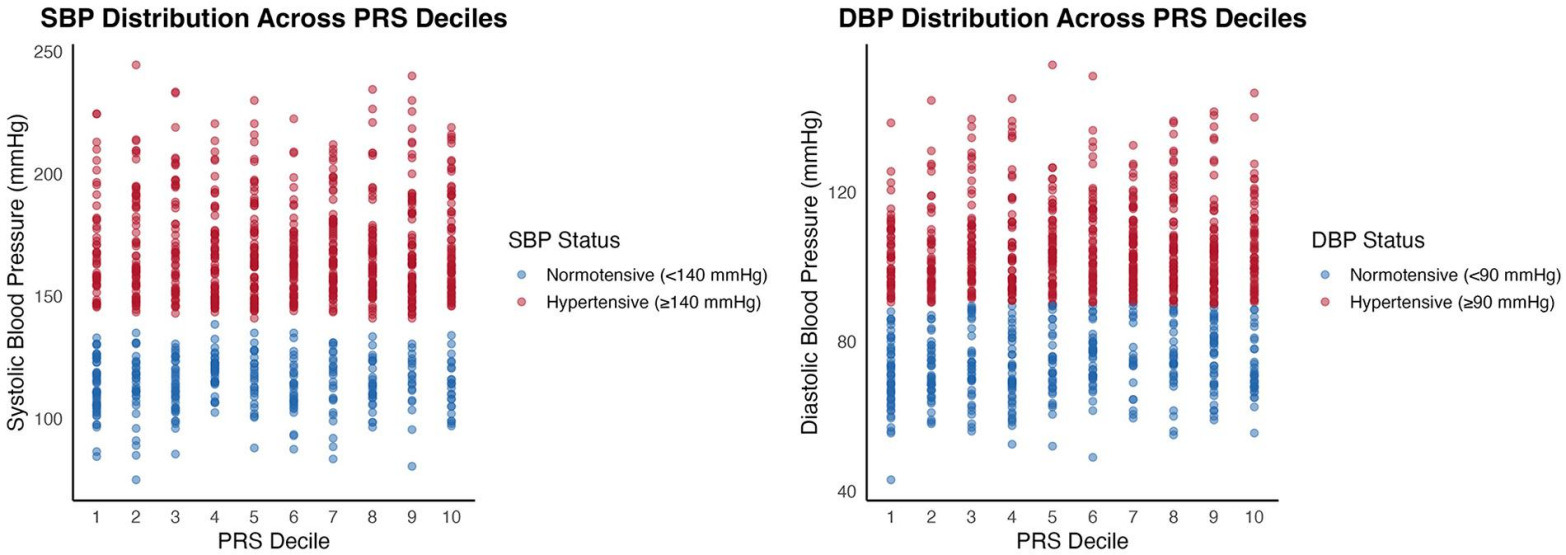
Distribution of individual SBP (left) and DBP (right) stratified by PRS decile and hypertension status. Scatter plots show individual SBP and DBP measurements across PRS deciles, with points colored by hypertension status (normotensive vs. hypertensive). Samples with SBP greater than or equal to 140 mmHg are colored red, and samples with SBP less than 140 mmHg are colored blue. Samples are colored red for DBP greater than or equal to 90 mmHg and blue for DBP less than 90 mmHg.

When comparing the first and last PRS deciles for SBP, the combined-pathway PRS distinguished between SBP groups with a moderate effect size (*t* = −3.38, *p* = 0.00087, Cohen's *d* = 0.49). The mean SBP for the first PRS decile was 141.2 mmHg (SD = 33.5), while the mean SBP for the last PRS decile was 157.0 mmHg (SD = 31.7) (Figures 1, 2).

For DBP, 1,163 independent SNPs remained after clumping. The optimal *p-*value threshold was 0.025, yielding a list of 70 SNPs for analysis, corresponding to 61 unique genes. The incremental increase in *R*² due to the DBP PRS was 0.0046 (1.4%, *p* = 0.012).

For DBP, comparing the first and last PRS deciles also yielded a significant distinction, albeit with a smaller effect size (*t* = −2.35, *p* = 0.0198, Cohen's *d* = 0.34). The mean DBP in the first PRS decile was 87.1 mmHg (SD = 19.2), while the mean DBP in the last decile was 93.4 mmHg (SD = 19.6) (Figures 1, 2).

**Figure 3:**
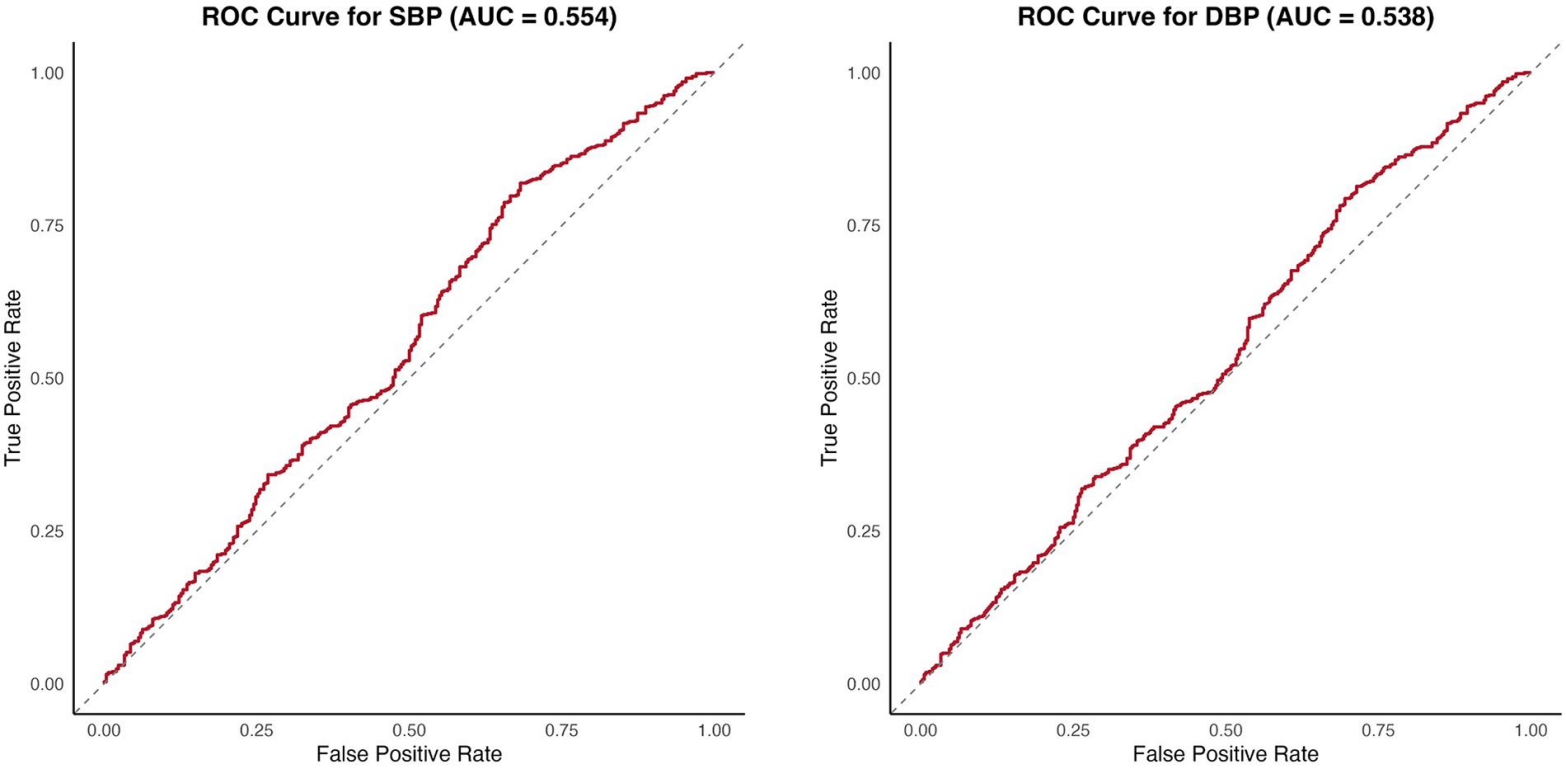
Area under the curve (AUC) for SBP polygenic risk scores (left) and DBP polygenic risk scores (right). Receiver operating characteristic (ROC) curves evaluate the ability of the aggregate pathway PRS to discriminate hypertension status (defined using standard SBP or DBP thresholds). AUC values are presented with corresponding 95% confidence intervals. Diagonal dashed lines indicate the null expectation. The 95% confidence interval (CI) for the SBP AUC is 0.5142–0.5944, and the 95% CI for the DBP AUC is 0.4996–0.5756.

To assess the predictive ability of each PRS for hypertension in a clinical context, we used the area under the receiver operating characteristic curve (AUC). Hypertensive patients were defined as those with systolic or diastolic blood pressures greater than 140 and 90 mmHg, respectively, or on antihypertensive medications. The combined-pathway PRS was not strongly predictive of clinical hypertension for either SBP (AUC = 0.596) or DBP (AUC = 0.557) in the target cohort (Figures 3).

In the MAP analysis, 1,158 independent SNPs remained after clumping. The optimal *p*-value threshold was 0.05, yielding 109 SNPs for analysis, corresponding to 92 unique genes. The incremental increase in *R*² due to the MAP PRS was 0.0031 (1.1% increase, *p* = 0.044). When comparing the first and last PRS deciles for MAP, the combined-pathway PRS showed a significant distinction with a moderate effect size (*t* = −3.17, *p* = 0.0018, Cohen's *d* = 0.46).

For pulse pressure, 1,149 independent SNPs remained after clumping. The optimal *p*-value threshold was 0.05, yielding 108 SNPs for analysis, corresponding to 89 unique genes. A statistically significant increase in *R*² due to the PRS was not observed in the pulse pressure analysis (*R*² = 0.0033, 5.6% increase, *p* = 0.066). When comparing the first and last PRS deciles for pulse pressure, the combined-pathway PRS did not reach statistical significance and suggested only a small effect size (*t* = −1.64, *p* = 0.103, Cohen's *d* = 0.24).

### Pathway-specific PRSs

**Figure 4:**
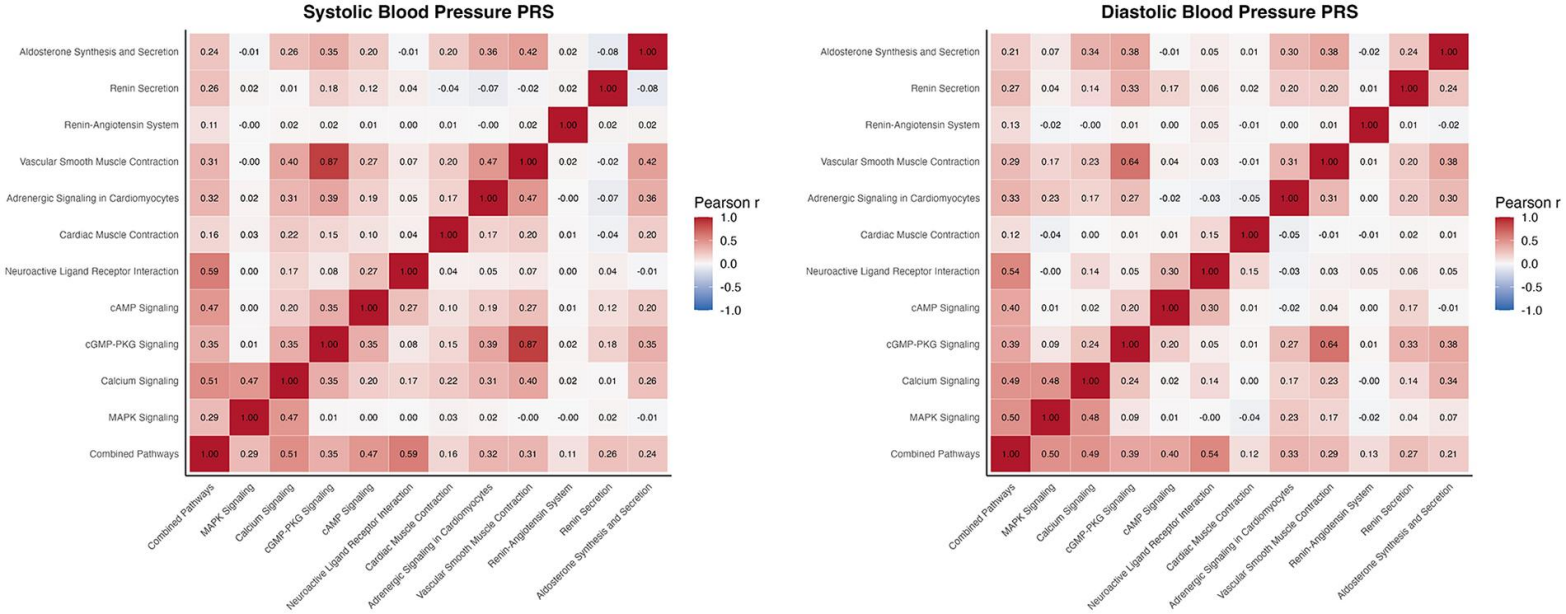
Pairwise correlation of pathway-specific PRSs for SBP (left) and DBP (right) in the discovery cohort. Heatmaps display Pearson correlation coefficients between pathway-specific polygenic risk scores (PRSs), including the aggregate (combined) pathway PRS, for systolic blood pressure (SBP) and diastolic blood pressure (DBP). Correlations were calculated using pairwise complete observations. Color intensity reflects the strength and direction of the correlations (blue = negative, red = positive), with numeric values shown within each cell. This panel illustrates the degree of shared genetic signal across pathway-specific PRSs and their relationship with the aggregate PRS.

In all, 11 pathway-specific polygenic risk scores were calculated for each of four blood pressure metrics. Due to overlapping SNPs and shared genetic architecture, moderate correlations were observed among PRSs from many pathways. For example, the PRSs for the vascular smooth muscle contraction pathway and the cGMP–PKG signaling pathway for DBP showed a correlation coefficient of 0.64. These pathways share 70 common genes, leading to a stronger correlation between their PRSs. Conversely, the renin–angiotensin system pathway shares no common genes with the cAMP signaling, mitogen-activated protein kinase (MAPK) signaling, and cardiac muscle contraction pathways, leading to correlation coefficients of 0.00, −0.02, and −0.01, respectively (Figures 4).

For DBP, the only pathway-specific PRS to produce a statistically significant incremental *R*^2^ was the cAMP pathway (incremental *R*^2^ = 0.0032, 1.0% increase, *p* = 0.036). Comparing the first and last PRS deciles for DBP, three pathways were able to best distinguish between high and low diastolic pressure, with comparable effect sizes: MAPK signaling pathway (*t*-statistic = −2.78, *p* = 0.0060, Cohen's *d* = 0.40), cAMP signaling pathway (*t*-statistic = −2.83, *p* = 0.0050, Cohen's *d* = 0.41), and adrenergic signaling in cardiomyocytes (*t*-statistic = −2.68, *p* = 0.0080, Cohen's *d* = 0.39).

None of the pathway-specific PRSs for SBP produced a significant increase in incremental *R*^2^, and no individual pathway-specific PRS predicted high systolic blood pressure when comparing first and last PRS deciles.

Pathway-specific PRSs were also calculated for pulse pressure and mean arterial pressure. Only the aldosterone synthesis and secretion pathway PRSs were significantly associated with increased pulse pressure when comparing first and last PRS deciles (*t*-statistic=−2.77, *p* = 0.0062, Cohen's *d* = 0.40). For mean arterial pressure, the adrenergic signaling in cardiomyocytes pathway was significantly associated with increased mean arterial pressure when comparing first and last PRS deciles (*t*-statistic = −2.50, *p* = 0.013, Cohen's *d* = 0.36).

## Discussion

In this study, a list of 1,149 unique genes was compiled from 11 different antihypertensive therapy-related pathways and used to calculate PRSs for different blood pressure metrics in a moderately sized, non-admixed West African cohort. In linear regression analyses, these PRSs were predictive of both elevated systolic and diastolic blood pressure. However, PRS analyses specific to individual pathways were not consistently predictive of blood pressure metrics.

Multiple groups have attempted to predict phenotypes, including quantitative measures of systolic and diastolic blood pressure, pulse pressure, and hypertension status, with successful demonstration that individuals carrying a greater burden of blood pressure-associated genetic variants of interest are more likely to have higher blood pressure (8, 32, 33). While several approaches to constructing PRSs have been developed since their original introduction, it is only recently that pathway-associated PRS methods have been used to study blood pressure (7). Pathway-specific PRSs can be considered a form of restricted, functionally informed form of polygenic risk scoring, in which functional annotation is derived from pathways targeted by therapeutic agents for the disorder. This approach offers unique utility in the study of hypertension, as multiple therapeutics, for example, ACEIs, angiotensin II receptor blockers (ARBs), and calcium channel blockers (CCBs), mediate their effects through well-defined pathways. These considerations helped inform the selection of pathways we studied, as they all play a role in one or multiple known mechanisms of action of the above medications.

### Calcium signaling, cardiomyocyte, and vascular smooth muscle contraction pathways

In this study, none of the pathway-specific PRSs for calcium signaling and muscle contraction were predictive of either systolic or diastolic blood pressure. Calcium signaling, through its downstream effects on various enzymes, including phospholipase C and ryanodine receptors, mediates contractility in both vascular and cardiac myocytes via the coordinated release of additional ionic calcium from the endoplasmic and sarcoplasmic reticula, respectively (34). This action is the primary target of calcium channel-blocking agents, e.g., amlodipine, which limit the re-entry of calcium into cells via the L-type calcium channel, thereby reducing the overall concentration of calcium in these tissues and contractility (34). Angiotensin receptor blockers also reduce vascular smooth muscle tone by blocking angiotensin II at the AT1 receptor, interfering with phospholipase C–inositol 1,4,5-trisphosphate– diacylglycerol (PLC–IP3–DAG) signaling, and inhibiting cellular proliferation via mitofusin-2 (35). A previous study showed that pathway-specific PRSs based on both cardiac muscle contraction and calcium signaling pathways were predictive of hypertension among individuals of European ancestry (7). However, in the study cohort, the genetic variants within these pathways may not have strong enough individual effects to be predictive in a polygenic risk score.

### Neuroactive ligand receptor interaction, cardiomyocyte adrenergic, and MAPK signaling pathways

In this study, the cAMP pathway PRS was predictive of elevated DBP. When comparing the first and last PRS deciles, both the cAMP and adrenergic signaling pathway PRSs could reliably distinguish between high and low DBP. Additionally, the first decile of the adrenergic signaling pathway PRS was predictive of elevated mean arterial pressure compared with the last decile. We found that the first PRS decile of the MAPK pathway was predictive of elevated DBP but not SBP compared with the last PRS decile.

Cardiomyocytes are subject to considerable sympathetic autonomic signaling by neuroactive ligands such as epinephrine and norepinephrine through both beta- and alpha-adrenergic receptors. Beta-adrenergic receptors stimulate cAMP production and downstream calcium release, ultimately resulting in stronger and faster contraction of the heart (34). Alpha-adrenergic receptors are similarly responsive to hormonal signaling through the PLC–IP3–DAG pathway. This mechanism is targeted by both ARB and CCB at the intracellular level, as previously described, and by beta-blocking agents extracellularly at neuronal–myocardial synapses (34).

Notably, CCB also inhibits the classical MAPK signaling pathway through the same mechanism. The MAPK signaling pathway is highly diverse, regulating many cellular functions from cell growth to tissue differentiation, and is sensitive to downstream effects of reduced calcium signaling through its relationship to the PLC–IP3–DAG pathway (36). Previous studies have linked multiple genes in the MAPK pathway to hypertension and beta-adrenergic receptor polymorphisms to blood pressure variability and alterations in cardiac output, findings that are in line with our results (37–39).

### Renin–angiotensin system, renin, and aldosterone secretion pathways

The pathway-specific PRSs related to the renin–angiotensin–aldosterone system were not predictive of either systolic or diastolic blood pressures on their own. However, the aldosterone synthesis and secretion pathway PRS was significantly associated with increased pulse pressure when comparing first and last PRS deciles (*t*-statistic=−2.78, *p* = 0.0062).

Alongside hormonal signaling from the central nervous system, much of blood pressure determination comes from precise renal sensing and regulation (40). In response to low sodium, low blood pressure, or beta-agonism, renin is released from the juxtaglomerular apparatus, leading to the downstream production and activation of angiotensin I and II and aldosterone (RAAS) (40). The resulting vasoconstriction and sodium salt/water retention increase blood pressure, although these pathways are frequently dysregulated in individuals with hypertension, secondary to overactivation of the RAAS system and faulty compensatory reactions in the central nervous system (41). ACEI and ARB are frequently chosen first-line agents for managing essential hypertension because of their efficacy against the actions of renin and aldosterone, primary mediators of blood pressure. It has been shown that higher plasma aldosterone concentrations are associated with greater pulse pressure, suggesting that elevated aldosterone levels may contribute to reduced arterial elasticity and increased pulse pressure (42–44). Similarly, a renin-angiotensin system pathway PRS has previously been shown to predict hypertension (7). However, this is the first instance in which an aldosterone synthesis and secretion pathway PRS has been linked to increased pulse pressure.

None of the individual pathway PRSs were independently significantly predictive of SBP. However, when genes from these pathways were combined to generate a single PRS, the resulting PRS could reliably predict elevated SBP.

In prior African-ancestry PRS studies, reported incremental *R*² for blood pressure traits typically range from <1% to <3%, similar to the values observed for the combined-pathway PRSs for SBP and DBP in this study (15, 16). The modestly increased *R*² values likely reflect the highly polygenic architecture of blood pressure traits. While these effect sizes are unlikely to be clinically actionable in isolation, they may still provide value for etiologic insight or population-level risk stratification.

The chief strength of the pathway-specific approach is that it enables more biologically interpretable PRSs. Specifically, by limiting the PRS gene sets to pathways targeted by common first-line antihypertensive medications, we gain better insight into clinically relevant genetic effects on blood pressure. For specific pathways, PRSs were moderately correlated due to overlapping SNPs. Pathways with higher correlation cannot be assumed to reflect pathway-exclusive genetic mechanisms, which can complicate biological interpretation. Accordingly, individual pathway-specific PRSs in this study are best interpreted as heuristic tools for exploring aggregation of genetic risk rather than definitive evidence of independent pathway-specific mechanisms. Overall, the poor predictability of individual pathway-specific risk scores in this study may be due to several factors.

Most notably, compared to large-scale biobank samples, we were limited by the size of our discovery (2,295 samples), validation (1,614 samples), and target (966 samples) cohorts. PRS analyses rely on large sample sizes because the effects of single nucleotide polymorphisms are often very minor and only become apparent at a very large scale. Similarly, hypertension is a complex multifactorial disease with genetic, epigenetic, and environmental influences. The genetic architecture of hypertension is highly complex, involving multiple genes with minor individual effects. This complexity can obscure the predictive power of PRS, especially when considering the polygenic nature of blood pressure regulation (45–47).

There exists an interplay between genetic and environmental factors in the development of hypertension, with some evidence that individuals genetically predisposed to high blood pressure may be more vulnerable to common modifiable risk factors (48). However, due to the cross-sectional nature of this study, we could not account for the myriad modifiable risk factors associated with hypertension risk (46, 49–51). Additionally, there can be substantial intraindividual variability in blood pressure measurements. A 2020 meta-analysis of ambulatory blood pressure reported 95% limits of agreement for daytime systolic BP ranging from −16.7 to 18.4 mmHg, which limits the interpretability of subtle changes (52). This variability can help explain the large standard deviations in both systolic and diastolic blood pressures observed among PRS deciles.

Blood pressure measurements for treated individuals were adjusted using fixed increments. While this method may introduce residual measurement error, prior studies suggest that genetic associations with continuous blood pressure traits are generally robust to reasonable variation in medication adjustment assumptions. Specifically, imputing BP for antihypertensive medications by adding a fixed value to SBP/DBP measurements does not significantly affect heritability estimates (53).

In contrast to continuous blood pressure traits, PRSs demonstrated limited discrimination for clinical hypertension status, with AUC values near 0.55–0.60. This likely reflects the inherent heterogeneity of phenotypes in binary hypertension definitions and reduced statistical power when modeling a dichotomous outcome. Since the diagnosis of hypertension depends on whether blood pressure exceeds a clinical threshold, rather than on underlying genetic architecture, converting continuous BP measurements to a binary hypertension outcome may result in loss of information. Individuals whose BP values fall just above and just below the cutoff are categorized differently despite having nearly identical genetic risk and BP levels. This threshold effect is particularly problematic, as genetic variants typically exert small effects on BP (approximately 1.0 mm Hg for SBP and 0.5 mm Hg for DBP per risk allele) (54). Continuous blood pressure measures may more directly capture underlying genetic liability and therefore represent a more appropriate outcome for PRS evaluation in this context.

Notably, some of our genotyping was performed using the Illumina Human Exome BeadChip v1.0, as described above. As this array is exome-wide rather than genome-wide, we do not account for contributions from non-coding genetic information, which is incompletely addressed by imputation. Additionally, this study is not intended to compare or evaluate different methods of PRS computation. We used the basic formula for PRS calculation implemented in PLINK because of its transparency, reproducibility, and robustness in moderate sample sizes. However, we understand that other methods, including PRSice, PRSice-2, PRS-CSx, LDpred2, and SbayesR, may yield different results and perhaps offer greater utility in studies where a predefined list of pathways was not already predetermined (13, 55–58). While newer alternative approaches may offer improved performance, particularly in larger or more diverse datasets, clumping-and-thresholding provides a conservative baseline appropriate for the present study.

Finally, while the relative genetic homogeneity of the Nigerian cohorts strengthens internal validity, it may limit generalizability to other African populations with distinct genetic backgrounds or to admixed populations. The study by Onyenobi et al., which developed genome-wide PRSs from African ancestry summary statistics derived from meta-analysis of multiple cohorts representing 21,912 individuals from across regions of Continental Africa, reported that the East Africa region showed the highest genome-wide PRS performance for SBP, DBP, PP, and hypertension, with *R*² values of 2.5%, 2.85%, 2.6%, and 0.95%, respectively (17). Whereas the three cohorts in this study were part of the meta-analyzed cohorts for only SBP and DBP in the study by Onyenobi et al., the objectives and approaches of the two studies differ and do not overlap. Replication of our findings in larger, regional, homogeneous African cohorts will be essential to assess their portability.

## Conclusion

Antihypertensive medications exert their effects through well-defined genetic pathways. We have shown that a combined PRS constructed from the genes in these pathways is predictive of both systolic and diastolic blood pressure in a West African-specific cohort. However, the relatively poor predictability of the pathway-specific PRS supports a broader influence of genetic, environmental, and epigenetic factors contributing to hypertension that cannot be captured by the pathway-specific PRS alone.

## Data Availability

The data analyzed in this study are subject to the following licenses/restrictions: the data presented in this study are restricted from publication due to ethical limitations but are available on request from the corresponding author. Requests to access these datasets should be directed to btayo@luc.edu.
